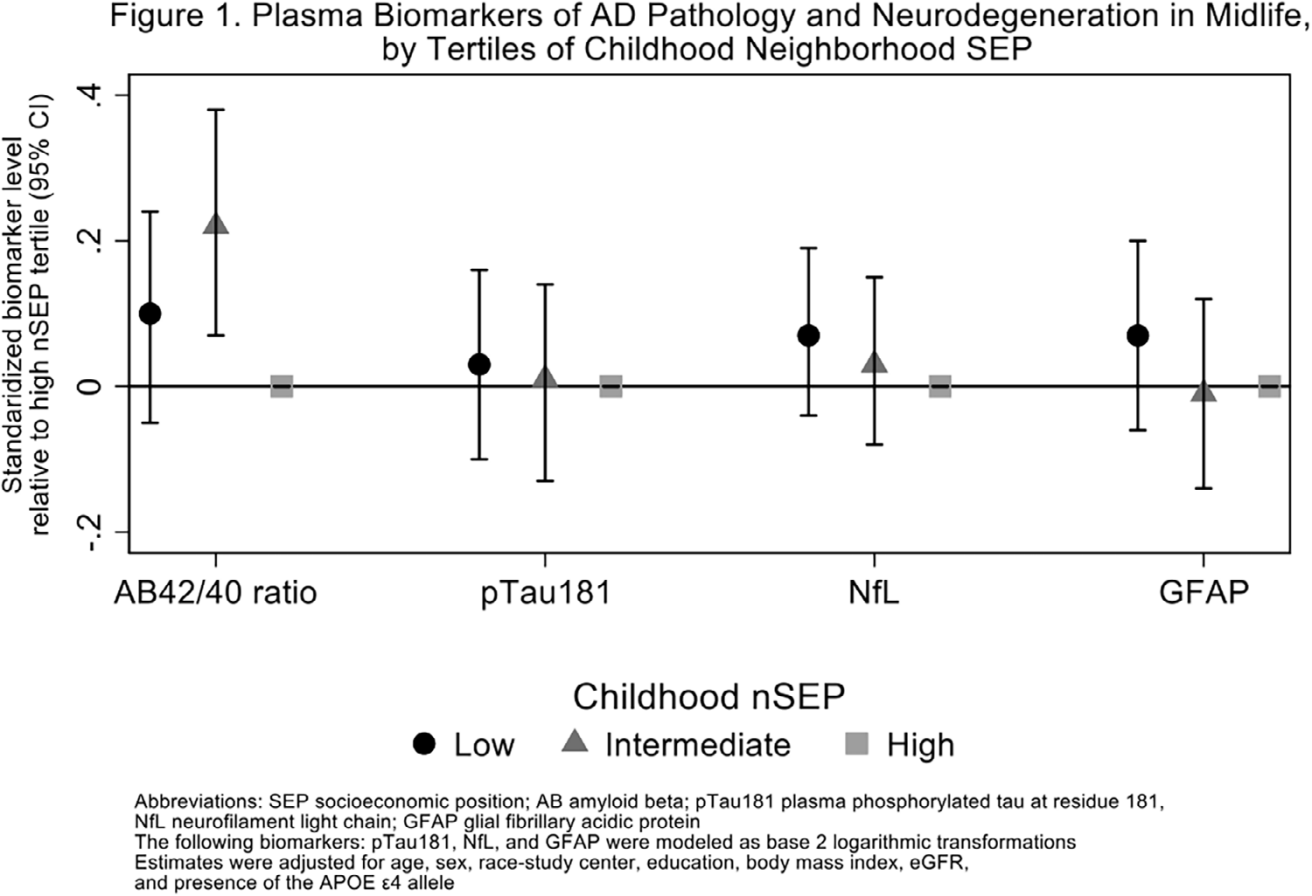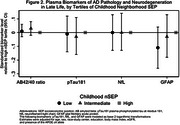# Childhood neighborhood socioeconomic position and levels of plasma markers of Alzheimer's disease pathology and neurodegeneration in adulthood; the Atherosclerosis Risk in Communities Study

**DOI:** 10.1002/alz70856_106661

**Published:** 2026-01-07

**Authors:** Anna M. Kucharska‐Newton, Jennifer Chen, James Russell Pike, Hannah Pleasants, Pamela L. Lutsey, Ganga Bey, Erin L. Abner, Eric A Whitsel, James D Stewart, Priya Palta

**Affiliations:** ^1^ University of North Carolina at Chapel Hill, Chapel Hill, NC, USA; ^2^ University of Kentucky College of Public Health, Lexington, KY, USA; ^3^ Departments of Population Health and Medicine, New York University Grossman School of Medicine, New York, NY, USA; ^4^ University of Minnesota School of Public Health, Minneapolis, MN, USA; ^5^ Department of Epidemiology and Environmental Health, University of Kentucky, Lexington, KY, USA

## Abstract

**Background:**

Early life exposure to adverse experiences correlates with poor health outcomes in adulthood. We therefore examined the association of childhood neighborhood socioeconomic position (nSEP) with midlife and late life levels of plasma‐based biomarkers of Alzheimer's disease (AD) pathology and neurodegeneration.

**Methods:**

The study population included 1362 Atherosclerosis Risk in Communities (ARIC) cohort participants (mean [SD] midlife age: 58.1 [4.8] years; 60.2% female; 26.4% Black) with amyloid‐β 42/40 (Aβ42/40), pTau‐181, neurofilament light chain (NfL), and glial fibrillary acidic protein (GFAP) ascertained at midlife (Visit 3; 1996‐1982) and older adulthood (Visit 5; 2011‐2013) using Quanterix Simoa assays. We used residential addresses at age 10 years to create a childhood composite of six, z‐scored U.S. Census‐based measures of nSEP: median household income; median value of owner‐occupied housing units; percent households receiving interest, dividend, or net rental income; percent adults with a high school degree; percent adults with a college degree; and percent adults in professional, managerial, or executive occupations. We used weighted, mixed‐effects models to estimate standardized log 2 transformed biomarker concentrations across tertiles of childhood nSEP adjusted for midlife age, race‐study center, sex, education, and presence of the APOE ε4 allele.

**Results:**

At midlife, participants who were in the lowest versus highest tertile of childhood nSEP had higher mean (SD) concentrations of pTau181(1.58 [0.94] vs. 1.53 [1.0], pg/ml), NfL (13.1 [7.4] vs. 12.1 [6.4], pg/ml), and GFAP (108.5 [64.9] vs. 103.9 [50.8], pg/ml.) Differences in biomarker concentrations between low and high childhood nSEP were attenuated in late life. The AB42/40 ratio at midlife and late life was comparable across the childhood nSEP tertiles. After covariate adjustment, midlife pTau181, NfL and GFAP concentrations were inversely associated with childhood nSEP (Figure 1). The association was marginally statistically significant for NfL (*p*‐trend=0.05). No associations were observed between childhood nSEP and levels of selected biomarkers ascertained in late life (Figure 2).

**Conclusion:**

Observed trend in the association of low childhood socioeconomic position with an adverse midlife, but not late life, profile of plasma‐based biomarkers of AD pathology and neurodegeneration warrants further study.